# Pro-social preference in an automated operant two-choice reward task under different housing conditions: Exploratory studies on pro-social decision making

**DOI:** 10.1016/j.dcn.2020.100827

**Published:** 2020-07-18

**Authors:** Jiska Kentrop, Aikaterini Kalamari, Chiara Hinna Danesi, John J. Kentrop, Marinus H. van IJzendoorn, Marian J. Bakermans-Kranenburg, Marian Joëls, Rixt van der Veen

**Affiliations:** a Dept. Translational Neuroscience, Brain Center Rudolf Magnus, University Medical Center Utrecht, Utrecht University, Utrecht, the Netherlands; b Dept. Psychology, Education and Child Studies, Erasmus University Rotterdam, the Netherlands; c Primary Care Unit, School of Clinical Medicine, University of Cambridge, United Kingdom; d Faculty of Behavioural and Movement Sciences, Vrije Universiteit Amsterdam, the Netherlands; e University of Groningen, University Medical Center Groningen, Groningen, the Netherlands; f Faculty of Social and Behavioural Sciences, Leiden University, Leiden, the Netherlands

**Keywords:** Rats, Complex housing, Pro-social decision making, Two-choice operant task, Food reward, Social development

## Abstract

In this study, we aimed to develop a behavioral task that measures pro-social decision making in rats. A fully automated, operant pro-social two-choice task is introduced that quantifies pro-social preferences for a mutual food reward in a set-up with tightly controlled task contingencies. Pairs of same-sex adult Wistar rats were placed in an operant chamber divided into two compartments (one rat per compartment), separated by a transparent barrier with holes that allowed the rats to see, hear, smell, but not touch each other. Test rats could earn a sucrose pellet either for themselves (own reward) or for themselves and the partner (both reward) by means of lever pressing. On average, male rats showed a 60 % preference for the lever that yielded a food reward for both themselves and their partner. In contrast, females did not show lever preference, regardless of the estrous cycle phase. Next, the impact of juvenile environmental factors on male rat social decision making was studied. Males were group-housed from postnatal day 26 onwards in complex housing Marlau™ cages that provided social and physical enrichment and stimulation in the form of novelty. Complex housed males did not show a preference for the pro-social lever.

## 1 Introduction

Pro-social behavior, defined as behavior that is aimed to benefit others, is a key element in many aspects of everyday life. It is proposed to be driven by the motivation to maintain social relations and is hypothesized to emerge from different forms of empathy, from emotional contagion (i.e. the ability to experience and share emotions) to more cognitive forms of empathy like adopting the other’s point of view (de Waal, 2008; De Waal and Preston, 2017). Empathy is based on personal embodied representations of emotions that may be mediated by the action of mirror neurons in both motor and emotional brain areas (Rizzolatti and Caruana, 2017). Empathy was traditionally thought to be a characteristic unique to human beings, but empathy for pain and fear and prosocial altruistic behaviors have also been found in both non-human primats and rodents (Chen, 2018; De Waal and Preston, 2017).

A number of studies have examined vicarious freezing behavior as an indicator of empathy in rodents (Atsak et al., 2011; Carrillo et al., 2019; Cruz et al., 2020; Han et al., 2020; Jeon et al., 2010; Pereira et al., 2012). In these studies usually an observer witnesses a demonstrator experience some kind of threat, most often a foot shock. It is shown that the observer “mirrors” the fear of the demonstrator and exhibits freezing behavior as well. Freezing seems to be even more intense in observers who themselves have previously experienced foot shock compared to naïve observers. Interestingly, some studies also show that this interaction is bidirectional as the behavior of the demonstrator is also affected by the behavior of the observer (Atsak et al., 2011; Han et al., 2020). In line with this, rats are willing to modify their behavior if this behavior is harming another rat, especially if the observer rat has prior shock experience (Hernandez-Lallement et al., 2020). In addition, rats that observe a food reward delivery to a conspecific have a modulated dopamine release in the ventral tegmental area, indicating that observed rewards are processed through similar neural pathways as direct rewards (Kashtelyan et al., 2014). The role of ultrasound vocalizations (USV’s) are import in the transmission of emotions in rodents. Conspecific USVs can evoke emotional contagion, both for positive and negative emotions, to change the affective states in receivers (Saito et al., 2016).

While emotional contagion in rodents has been widely observed, only recently studies started reporting that smaller brained animals also show pro-social decision making, that would require higher cognitive processes (Bartal et al., 2014, 2011; Hernandez-Lallement et al., 2016; Sato et al., 2015; Sivaselvachandran et al., 2018; Stetzik et al., 2018). Various types of tasks have been developed to study the relationship between empathy and pro-social decision making in rodents. For example, Bartal et al. (2011) showed that rats liberated a conspecific that was trapped in a tube, they did not show this behavior when the tube was empty or contained an object. Similarly, in a study from Sato and colleagues (2015) rats learned to open a door in order to liberate a soaked conspecific from a wet area. This behavior was observed mainly when the conspecific was stressed and was consistent even when the rats were presented with the choice to open another door in order to obtain food. In other studies, rats were also found to cooperate in a task that involved pulling a baited stick that produced food for a partner (Rutte and Taborsky, 2007; Schneeberger et al., 2012). Not only did they reciprocate help to familiar partners that helped them before (direct reciprocity), they were also more willing to cooperate with unfamiliar partners if they received help before (generalized reciprocity) (Rutte and Taborsky, 2007). Moreover, in a rodent version of the Prisoner’s Dilemma game rats understood the payoff matrix of the game and were capable of optimizing their cooperative strategy based on the behavior of their partner (Viana et al., 2010).

Another example of a pro-social decision making task, studied in a variety of animals, is the pro-social (two-)choice task (Silk et al., 2005). In this task, the test subject has to perform an act (i.e. handing over a token or choosing a side in a maze) to earn a food reward, and has two options: option A leads to a food reward only for the test subject (selfish choice) and option B leads to a food reward for both the test subject *and* partner (pro-social choice). In this task the effort required and the pay-off for the test subject is equal for both choices (i.e. the pro-social choice provides a benefit for the other at no cost of the test subject). The pro-social two-choice paradigm has been mostly tested in primates (Burkart et al., 2007; de Waal et al., 2008; Horner et al., 2011), but studies in birds (Brucks and von Bayern, 2020; Di Lascio et al., 2013; Massen et al., 2015; Palagi et al., 2016; Péron et al., 2013; Schwab et al., 2012) and rats (Hernandez-Lallement et al., 2016a, 2015; Márquez et al., 2015) have been reported as well (see Marshall-Pescini et al., 2016 for an elaborate review).

Thus far, with the non-costly pro-social two-choice task, pro-social preferences have been established in multiple species. Yet, test conditions played a major role in whether or not animals displayed pro-social behavior in a laboratory test setting; strict control of environment and test contingencies are clearly essential (Cronin, 2012; House et al., 2014; Marshall-Pescini et al., 2016). Many factors, e.g. familiarity between test subject and partner (Burkart et al., 2007; de Waal et al., 2008), sex (of the partner) (Burkart et al., 2007; Schwab et al., 2012), body weight differences between test subject and partner (Hernandez-Lallement et al., 2015), and partner behavior during the task (Horner et al., 2011; Márquez et al., 2015; Schwab et al., 2012) were shown to influence decision making in this paradigm (Cronin, 2012; Marshall-Pescini et al., 2016).

Rearing environment and previous (social) experiences probably also affect the development of social decision making. Environmental factors influence brain development, especially during sensitive periods, including (early) adolescence (Marco et al., 2011; Sandi and Haller, 2015). Evidence for this stems from studies in both humans and rodents that link adverse events in adolescence to the development of aberrant social behavior and increased risk for psychopathology (Fuhrmann et al., 2015; Tzanoulinou and Sandi, 2016). In rodents, the impact of environmental influences on brain development can be studied by manipulating the rearing conditions. Enrichment of housing conditions (both physical and social) has been shown to positively affect social behavior. For example, rats living in a socially -but not physically- enriched environment showed more social approach behavior in response to 50 kHz affective calls in a radial arm maze (Brenes et al., 2016). Recently, our lab has shown that housing of rats in a complex living environment with physical, social and novelty enrichment from early adolescence onwards has mixed effects on social behavior (Kentrop et al., 2018). In adolescence, complex housed male and female rats showed shorter latencies to engage in social play; and males showed more social play behavior. However, adult males (but not females) showed less social interest in a three-chamber social approach task.

To further investigate the role of a complex living environment from early adolescence onwards in later pro-social behavior, we developed a new, fully automated, operant version of the pro-social two-choice task for rats. In this test, pairs of rats are placed in an operant chamber, divided into two compartments by a transparent barrier with holes that allows the rats to see, hear, smell, but not touch each other. The test rats can earn food rewards for themselves only (own reward: OR) or for themselves and the partner (both reward: BR) by means of lever pressing. This simplified operant design eliminates the direct influence of experimenters during the test and maximizes standardization of task variables. We provide a task where the actor rat (test rat) can adapt its behavior to provide a reward to a conspecific rat, while its behavior is not necessarily dependent on the actions performed by this conspecific. For instance, the test rat does not have to wait until the partner rat enters a compartment (compare Hernandez-Lallement et al., 2015; Márquez et al., 2015) before continuing the task. Moreover, since the location of the reward delivery in our task is the same for rewards obtained on the BR or OR levers (that is, in the middle) partner rats cannot actively demonstrate a preference for the BR side (compare Márquez et al., 2015). In the present study we performed the task with both males and females. Moreover, the impact of early life environmental rearing conditions on pro-social decision making was studied in males. Male rats were housed from postnatal day 26 onwards in groups of 10 in complex housing Marlau™ cages that provided social and physical enrichment and were next tested in the pro-social two-choice task in adulthood.

## 2 Materials and methods

### 2.1 Animals

Male and female Wistar rats used for breeding were obtained at 8–10 weeks of age (Charles River Laboratories, Arbresle, France). All experimental rats were bred in-house (see section 2.2). Rats were kept in type IV Makrolon cages (37 × 20 × 18 cm) in temperature (21 °C) and humidity (55 %) controlled rooms with a 12 h light–dark cycle (lights on from 08:00−20:00) during breeding, and a reversed cycle (lights on from 20:00−08:00) from postnatal day (PND) 21 onwards. Food and water were available ad libitum until the start of behavioral testing. The male complex housing experiment was performed to study the effect of environment on pro-social behavior (*n* = 33 standard housed pairs versus *n* = 29 pairs complex housed). The female experiment was conducted with *n* = 18 female pairs. Rats were tested in adulthood (PND 90+) in pairs; one acting rat, referred to as *test rat*, and one stimulus rat, referred to as *partner*. All males participated either as test rat or partner, whereas females conducted the experiment once as test rat and once as partner. Once per week cages were cleaned and general health status was checked. Standard housing cages were provided with a woodblock as standard cage enrichment. Experiments were approved by the Central Authority for Scientific Procedures on Animals in the Netherlands (CCD project AVD115002016644). Animal care was conducted in accordance with the EC Council Directive of November 1986 (86/609/EEC).

### 2.2 Breeding and complex housing environment

Breeding started after the rats had been familiarized with our animal facility for at least two weeks. Two females were paired with one male for 10 days. After separation from the male, females stayed together for another week and were then individually housed to prepare for birth. Paper towels were provided to the mothers as nesting material. The day of birth was considered PND 0. At PND 3 the sex of the pups was determined. Litters were culled to a maximum of 10 pups or supplemented to a minimum of 6 by adding pups from culled litters. All litters contained at least two pups from each sex. Weaning was on PND 21. From weaning onwards, standard housed rats (males and females) were pair-housed in type IV Makrolon cages (37 × 20 × 18 cm). In the complex housing experiment, animals were semi-randomly assigned to the experimental or control groups, with the constraint of a maximum of two rats from the same litter assigned to either the standard condition or one of the two complex cages.

**Fig. 1:**
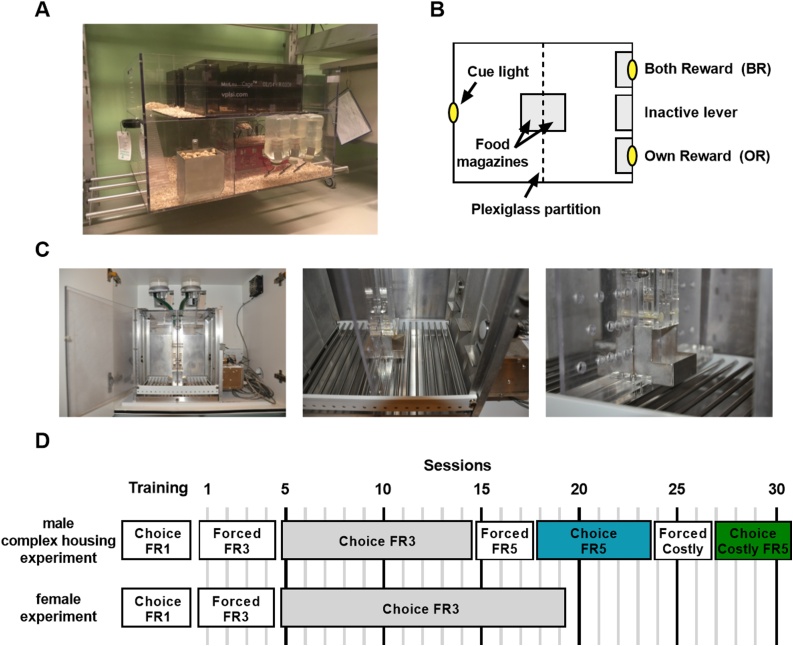
(A) Complex housing Marlau™ cage for rats (manufactured by Viewpoint, France). (B) Schematic representation of the pro-social two-choice set-up in an operant chamber (29.5 × 23.5 × 27.3 cm, Med Associates, St. Albans, VT, USA). The chamber is divided in two equal sized compartments using a transparent Plexiglas partition with holes (6 mm in diameter) that allows the rats to see, hear and smell, but not touch each other. The partner rat compartment (left side) contains only a cue light, while the test rat compartment (right side) is equipped with 3 retractable levers on the right wall. Sucrose pellets are dispensed in metal pellet magazines that are attached to the Plexiglas partition. The pellet magazines are located opposite to each other (separated only by the Plexiglas partition) and designed to ensure that rats can see and hear pellets being delivered to the other rat. (C) Photos of the pro-social two-choice set up of the operant chamber (left), test rat compartment (middle) and transparent Plexiglas partition and pellet magazines (right). (D) Schematic overview of behavioral protocols used in the different experiments.

Rats assigned to complex housing (CH) were first housed in type IV Makrolon cages with 3–4 males from PND 21–26. On PND 26, rats were transferred to Marlau™cages (Viewpoint, Lyon, France, Fig. 1A), housing 10 males per cage. The CH rats remained in the Marlau cages throughout the experiment. Marlau cages (60 × 80 × 51 cm) have two floors and provide a complex and challenging environment for the rats (Fares et al., 2013). The first floor contains a big compartment with three running wheels, a shelter, ad libitum access to water, two woodblocks, and a climbing ladder to the second floor, where a maze has to be passed to gain access to a tube leading to the food compartment on the first floor. Via a one-way passage rats could regain access to the bigger first floor compartment. The maze was changed once per week (alternating between 12 different configurations), assuring novelty and sustained cognitive stimulation. Territorial dominance was avoided by the presence of two openings on each side of the maze. A more detailed description of the experimental setup is given elsewhere (van der Veen et al., 2015).

### 2.3 Boldness test

To determine which rats within a male pair would become test rat or partner in the two-choice task, boldness was measured. The partner rat was always the same familiar cage mate. Boldness was measured by opening the lid of a cage half-way and measuring for each rat how much time was needed to rear and place at least 1 paw on the edge of the cage. This test was repeated 3 times on separate occasions and rats were ranked based on their cage emergence latency. Rats with the highest boldness rank were selected as the test rat in a later performed liberation task (data not shown here) in which boldness is a selection criteria (Bartal et al., 2011), the rats with lowest boldness scores were test rats in the two-choice task. Females were not tested in the liberation task further on and acted in the 2-choice task as both test rat and partner rat.

### 2.4 Pro-social two-choice task

#### 2.4.1 Apparatus

The two-choice task was conducted in adapted operant chambers set-up (30.5 × 24.1 × 21 cm, Med Associates, St. Albans, VT, USA). Experimental contingencies were controlled and data were collected using MED-PC version 14.0 (Med Associates). The operant chambers were enclosed in larger boxes equipped with exhaust fans that assured air renewal and masked background noises. Each chamber was divided in two equal sized compartments using a transparent Plexiglas partition (6 mm thick) with holes (6 mm in diameter) that allowed the rats to see, hear and smell, but not touch each other (see Fig. 1B and C). Two pellet dispensers containing sugar pellets (45 mg, Formula P; Bio-Serv, UK) were located on top of the chamber, one above each compartment. The test rat compartment (right side) was equipped with 3 retractable levers on the right wall: 1) an ‘Own Reward’ (OR) lever + cue light through which the test rat could earn a sucrose pellet reward without reward for the partner, 2) an inactive lever in the middle and 3) a ‘Both Reward’ (BR) lever + cue light through which the test rat could earn a sucrose pellet for both itself and the partner rat. Presses on the inactive lever were registered but had no consequences for the test rat. The position of OR and BR levers was counterbalanced across the boxes. The partner rat compartment (left side) contained only a cue light that lit up when the test rat earned a reward for both by pressing on the BR lever. Chamber light was turned off, so animals were in the dark, but could see each other during reward delivery when cue light lit up. Sucrose pellets were dispensed in two metal pellet magazines in the middle. The pellet magazines were located opposite to each other (separated only by the Plexiglas partition) and designed to ensure that rats could see and hear pellets being delivered to the other rat.

#### 2.4.2 Temporary mild food restriction and sucrose pellet habituation

One week before the start of behavioral testing, animals were mildly food restricted (3–4.5 g chow/100 g body weight) to attain 90–95 % of their free fed bodyweight. Animals were weighed twice per week to monitor body weight. The week before testing rats received sucrose pellets in the home cage on three occasions to habituate to the taste of the pellets. Bodyweight loss from the start of the food restriction period until the start of FR3 testing and bodyweight loss during FR3 testing was used in the bodyweight loss analyses.

#### 2.4.3 Behavioral procedure

Fig. 1D depicts a schematic overview of the behavioral protocols used, both experiments used a design with forced alternation sessions followed by free choice test sessions as described below.

*Habituation:* Both rats of a couple were habituated to the set-up for 15 min during which none of the levers were extended and a sucrose pellet was delivered to both rats every minute.

*General trial design:* All sessions lasted 30 min and rats were tested once (females) or twice (males) per day for at least 5 days per week. If a test rat earned a pellet by pressing the OR lever, all levers retracted, a cue light above the OR lever turned on for 20 s to signal reward delivery; a sucrose pellet was dispensed in the pellet magazine of the test rat. If the test rat pressed the BR lever, all levers retracted, the cue light above the BR lever and the cue light in the center of the partner rat compartment turned on for 20 s and a sucrose pellet was delivered in both pellet magazines. In both cases, after an inter-trial interval of 20 s all levers were extended again and a new trial started. Sucrose pellets for the partner rat were delivered without delay, while the sucrose pellets for the test rat were delivered with a delay of 3 s in both conditions (OR and BR) to ensure that the test rat could see and hear the reward for the partner being delivered before consuming its own reward.

*Fixed ratio (FR) 1 training:* Rats were trained on a fixed ratio 1 (FR1) protocol where all levers were extended and each lever press on the OR or BR lever resulted in the delivery of a sucrose pellet for self (OR) or both (BR). Rats were trained on this protocol until the test rat made at least 30 responses within one session (thus earned at least 30 pellets).

*Forced alternation*: Before every block of ‘free choice’ testing, rats were given 3 or 4 sessions of forced alternation to ensure that test rats sampled both active levers under the new contingencies. During forced alternation only the OR or BR lever was present together with the inactive control lever in the middle. The presentation of OR or BR lever alternated in blocks of 5 min in an A-B-A-B-A-B schedule, with random allocation of the presented active lever in the first 5-min block.

*Free choice FR3 and FR5 testing:* During free choice testing all levers were available and test rats could earn one sucrose pellet by pressing the OR or BR 3 times (free choice FR3) or 5 times (free choice FR5 or free choice FR5 ‘costly’ [see below]). For females, 15 free choice FR3 sessions were conducted to ensure that BR preference data was available for each phase of the estrous cycle.

*‘Costly’ pro-social protocol*: To challenge the pro-social BR preference of rats, the ‘cost’ of the reward associated with the BR lever for the test rat was increased by increasing the sucrose reward delivery latency (i.e. rats had to wait longer for the sucrose pellet). The reward delivery delay for the BR lever for the test rat was increased from 3 s to 5 s, while the reward delivery delay for the OR lever remained 3 s. The reward delay for the partner rat for the BR lever remained 0 s.

#### 2.4.4 Behavioral parameters

Measured parameters included number of presses on the BR lever, OR lever and middle inactive lever, and number of rewards obtained by the test rat and partner. For each rat a pro-social preference score (i.e. the percentage rewards obtained on the BR lever) was calculated per session based on activity on the two active levers: pro-social preference score = (number of BR choices/number of BR + OR choices)*100 %). The inactive middle lever served as a control for lever press acquisition and because rats could not earn a sucrose pellet upon responding to this lever it was not included in the preference score.

### 2.5 Estrous cycle determination in females

To determine the circulating levels of female sex hormones throughout the estrous cycle, vaginal smears were obtained directly after each test session. Vaginal smears were collected by inserting the head of a sterile plastic smear loop (1 μL, VWR, the Netherlands) and gently swabbing the vaginal wall. The collected cells were transferred to a drop of water on a glass microscope slide, air-dried and stained with a 5 % Giemsa (Sigma-Aldrich, The Netherlands) in water solution. Microscopic evaluation of the cells present in the vaginal smears was performed by a trained observer and was used to determine the phase of the estrous cycle (Cora et al., 2015; Goldman et al., 2007). The following four parameters were estimated: the relative amount of cells present (on a scale from 1 to 5), and the percentage of nucleated cells, anucleated cells and leukocytes. Based on these four parameters and taking into account all days, smears were assigned as pro-estrus, estrus or metestrus-diestrus, according to (Cora et al., 2015). Based on this evaluation, behavioral data was split into two groups; pro-estrus/estrus (P/E) and metestrus/diestrus (M/D), with high and low levels of circulating female hormones, respectively. Data from two females was excluded from the analysis due to persistent metestrus/diestrus smears lasting for more than 12 days, indicating a pseudopregnancy (Cora et al., 2015; Goldman et al., 2007).

### 2.6 Statistical analysis

Statistical analyses were performed using SPSS for windows version 26 (IBM, United States). Outlying scores (3.29 standard deviations below or above the mean) were winsorized, i.e. substituted with a value just below or above the next extreme score, respectively (Tabachnick and Fidell, 1996), to mitigate excessive influence of outliers without excluding subjects. In total 9 outliers in 6 different variables were winsorized.

To analyze task acquisition and stability in responding, repeated measures ANOVAs were conducted on the number of rewards earned and pro-social BR preference of the test rat over the course of free choice FR3 test sessions. If Maughly’s test of sphericity was violated, a Green-house Geisser correction was performed on the degrees of freedom. To test if rats chose the BR option more often than chance, the average percentage of rewards obtained on the BR side (i.e. percentage pro-social BR choices) during the last three sessions of a protocol was tested against 50 % with a two-tailed one-sample Student’s *t*-test. A one-way ANOVA was conducted with pairwise comparisons (Bonferroni correction for multiple testing) on the number of lever presses on the inactive, OR and BR lever. For the females, all behavioral data from session 6 onwards obtained during pro-estrus and estrus was averaged to provide one data point per female and likewise for data obtained during metestrus and diestrus. To test differences in BR preference in these different phases of the estrous cycle, a paired-samples Student’s *t*-test was used. To determine whether the order in which the females were tested (i.e. first as test rat or partner rat) affected their behavior an independent samples *t*-test was performed for both phases in the cycle. As a proxy for the influence of dominance on the willingness to share food with a partner, we used the difference in bodyweight (i.e. between the test rat and partner) as an indicator of dominance (Boreman and Price, 1972; Militzer and Reinhard, 1982; Smith et al., 1994). The correlation between bodyweight difference and BR preference was computed using Pearson’s *r*. Likewise, the correlation between body weight loss of both rats and BR preference was computed.

For the complex housing experiment, repeated measures ANOVAs with protocol (i.e. the average of the last 3 sessions for each protocol) as within-subject factor and housing condition (complex vs standard) as between-subject factor were conducted to analyze the effect of changing the protocol on BR preference. Where appropriate, partial eta squared *ƞ_p_^2^*, Cohen’s d_z_, Hedges’s g_s_ or g_av_ were used to report effect sizes according to (Lakens, 2013).

## 3 Results

Several pilot experiments were conducted to develop the task and optimize behavioral protocols. All pilots (conducted with males) contained a training phase and a free choice FR3 phase. When combined, clear differences in responding to the inactive and active levers were observed and we found an overall preference of 60 % for the BR lever (see Fig. S1). Over the course of FR3 testing the animals optimized performance and increased the number of rewards obtained within a session. Based on these pilot results, we reverted to the fully powered experiment addressing our actual research question.

### 3.1 Prosocial preference in males and housing effects in the 2-choice task

**Fig. 2:**
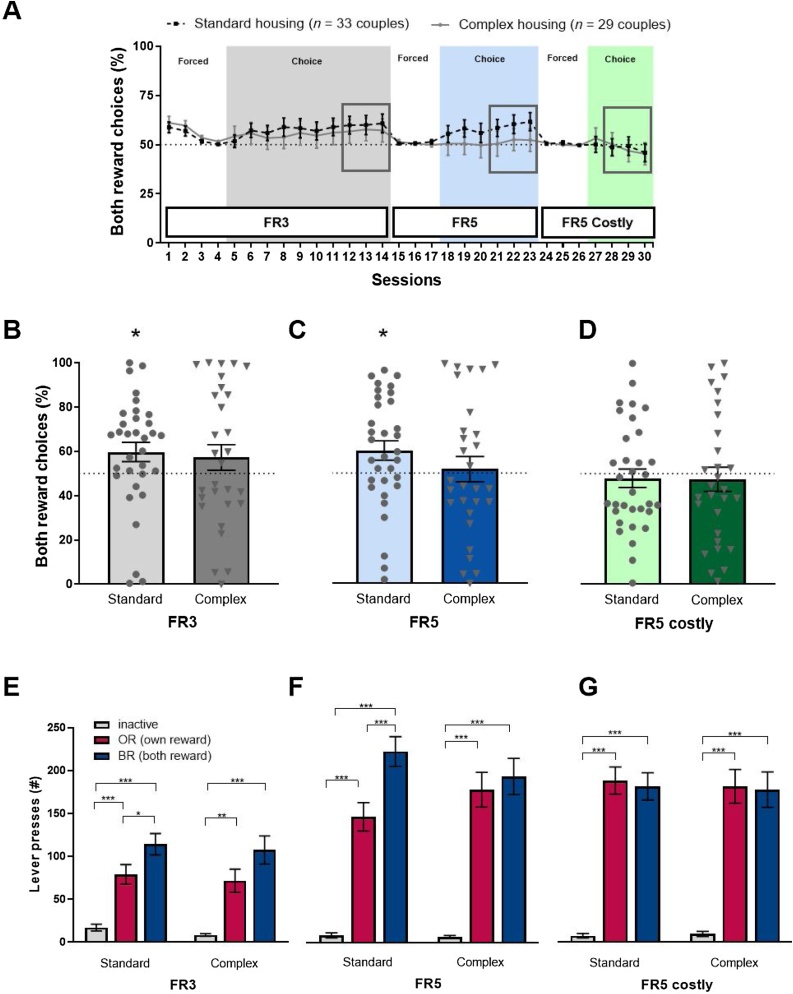
Pro-social Both Reward (BR) preference comparing complex (n = 29 couples) to standard housed males. (A) BR preference represented as the percentage of BR choices per session on different protocols; FR3 (grey), FR5 (blue) and FR5-Costly protocol (green). ‘Forced’ indicates forced alternation training and ‘choice’ indicates a free choice protocol. The last 3 sessions on each protocol were averaged for (B-D) Average BR preference on free choice FR3 (B), free choice FR5 (C), and free choice FR5-Costly (D). *indicates a significant difference from 50 % chance. (E-G) Average number of lever presses on the inactive, own reward (OR), and both reward (BR) levers, in the free choice FR3 (E), free choice FR5 (F) and free choice FR5 costly (G) protocols. Standard housing *n* = 33 couples, complex housing *n* = 29 couples. *p < .05, **p < .01, ***p < .001. (For interpretation of the references to colour in the Figure, the reader is referred to the web version of this article).

Like we observed in the pilot experiments, performance of both standard and complex housed males in terms of number of rewards earned improved over the course of FR3 testing (Rewards *F*(3.1, 182.1) = 4.73, *p* < .01, *ƞ_p_^2^* = .07, Rewards*Housing *F*(3.1, 182.1) = 2.57, *p* = .056, *ƞ_p_^2^* = .04 (graph not shown) and BR preference on the free choice FR3 protocol was stable (Sessions *F*(4.3, 256.4) = 1.40, *p* = .23, *ƞ_p_^2^* = .02, Sessions*Housing *F*(4.3, 256.4) = 0.44, *p* = .80, *ƞ_p_^2^* = .007, Fig. 2A). The average behavior of the last 3 sessions was used for further analysis (Fig. 2B-D).

The standard housed animals showed an overall 60 % preference for the BR lever on FR3, which was significantly different from chance (*t*-test against 50 %: *t*(32) = 2.22, *p* < .05, *d*_z_ = 0.38), while complex housed animals did not show a preference different from chance (*t*-test against 50 %: *t*(28) = 1.24, *p* = .22, *d*_z_ = 0.22, Fig. 2B). However, the level of BR preference itself did not differ between groups (standard vs complex *t*(60) = .35, *p* = .72, *g_s_* = 0.08). For both groups, the number of lever presses on free choice FR3 was significantly lower on the inactive versus active levers (standard: *F*(296) = 23.8, *p* < .001, *ƞ_p_^2^* = .33; complex: *F*(284) = 16.5, *p* < .001, *ƞ_p_^2^* = .28, pairwise comparisons for inactive lever vs BR or OR p < .001 or p < .01 in both housing conditions). However, the preference towards the BR versus the OR lever was significant only for the standard housed group (pairwise comparisons for BR vs OR standard: *p* < .05; complex: *p* = .13, Fig. 2E).

Increasing the fixed ratio from FR3 to FR5 in the complex housing experiment, requiring more effort from the test rat to obtain a food reward, did not affect preference in either housing condition (Protocol *F*(160) = 0.92, *p* = .34, *ƞ_p_^2^* = .02, Protocol*Housing *F*(160) = 1.25, *p* = .27, *ƞ_p_^2^* = .02, with no overall housing effect: Housing *F*(160) = 0.68, p = .41, *ƞ_p_^2^* = .01). Similarly to the FR3 protocol, standard housed animals on average showed a 60 % preference for the BR choice on the FR5 protocol, being higher than chance level (*t*-test against 50 %: *t*(32) = 2.29, *p* < .05, *d*_z_ = 0.39), while complex housed animals did not differ from chance (*t*-test against 50 %: *t*(28) = 0.31, *p* = .76, *d*_z_ = 0.03, Fig. 2C). Note that in the forced sessions in-between FR3 and FR5 only one active lever (OR or BR) is available at a time and animals sample both levers equally; standard housed animals returned to an overall pro-social choice when both active levers were available in the choice sessions. The number of lever presses on the inactive versus active levers in free choice FR5 was significantly lower for both groups (standard: *F*(296) = 60.7, *p* < .001, *ƞ_p_^2^* = .56; complex: *F*(284) = 37.5, *p* < .001, *ƞ_p_^2^* = .47, inactive lever vs BR or OR p < .001 in both housing conditions). As in the FR3 protocol, the preference of the BR versus the OR lever was significant only for the standard housed group (BR vs OR standard: *p* < .001; BR vs OR complex: *p* = 1.00, Fig. 2F).

For the FR5-Costly protocol, the reward delivery latency for the test rat was extended to 5 s for the BR lever (versus 3 s for the OR lever). For practical reasons this protocol was only performed for 4 days, but what can be seen is that overall animals did not show a preference for the BR lever in the free choice FR5 costly session, i.e. both groups performed at chance level (Fig. 2A and D). The costly condition indeed affected BR preference (Protocol *F*(160) = 18.58, *p* < .001, *ƞ_p_^2^* = .24), with a stronger decrease in responding for the standard housed animals (Protocol*Housing *F*(160) = 4.24, p < .05, *ƞ_p_^2^* = .07). In this protocol, neither standard nor complex housed animals differed in BR preference from chance level (*t*-test against 50 %, standard *t*(32) = -.50, *p* = .62, *d*_z_ = -0.08; complex: *t*(28) = −0.47, *p* = .64, *d*_z_ = -0.08), Congruently, the number of lever presses on the OR vs BR levers was not different for either housing condition, while the inactive lever was still clearly different from both active levers (standard: *F*(296) = 61.7, *p* < .001, *ƞ_p_^2^* = .56, complex: *F*(284) = 35.4, *p* < .001, *ƞ_p_^2^* = .46. Inactive vs OR or BR *p* < .001 for both conditions and BR vs OR *p* = 1.00 for both conditions, Fig. 2G).

There was no relation between body weight difference within a pair (as a proxi for dominance) and BR preference in either group (standard housed: *r* = .25, *p* = 0.25, Mean ± SEM bodyweight for test rats: 375.2 ± 14.2, Mean ± SEM bodyweight for partners: 372.8 ± 13.8, body weight difference range: 74.9–138 %; complex housed: *r* = 0.11, *p* = 0.65, Mean ± SEM bodyweight for test rats: 404.8 ± 14.3, Mean ± SEM bodyweight for partners: 397.1 ± 11.8, body weight difference range: 90–123 %). Throughout the period of behavioral testing, all animals were mildly food restricted. BR preference was not associated with percentage body weight loss from the start of food restriction to the start of FR3 testing (standard housed: test rats *r* = −0.06, *p* = 0.80 and partners *r* = 0.08, *p* = 0.73; complex housed: test rats *r* =0.32, *p* = 0.18 and partners *r* = 0.35, *p* = 0.14). There was a negative correlation between BR preference and body weight loss of the partner within the FR3 session, that was significant in the complex housed group (standard housed: test rats *r* = -0.17, *p* = 0.42 and partners *r* = −0.36, *p* = 0.08; complex housed: test rats *r* = 0.28, *p* = 0.25 and partners *r* = -0.60, *p* = 0.01). Partners that received less sucrose pellets in the test were lighter at the end of FR3 testing, indicating that the behavior of the test rat had a direct and considerable impact on the partner.

### 3.2 Pro-social preference in the two-choice task in females

Like in males, the percentage of BR choices over the course of testing was stable at group level (*F*(14,238) = 0.56, *p* = .67, *ƞ_p_^2^* = .03), and there was an increase in the amount of rewards earned per session (*F*(14,238) = 3.65, *p* < .01, *ƞ_p_^2^* = .18, graphs not shown).

**Fig. 3:**
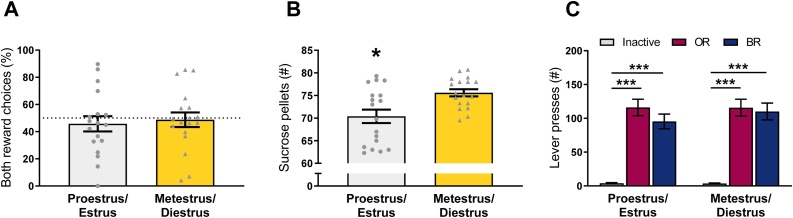
Pro-social Both Reward (BR) preference in females in different phases of the estrous cycle; proestrus/estrus (P/E, i.e. high levels of circulating female hormones) and metestrus/diestrus (M/D, i.e. low circulating hormone levels). (A) BR preference on a free choice FR3 protocol represented as the percentage of BR choices. (B) Number of rewards earned per session. (C) Number of responses on the inactive (grey), OR (pink), and BR (blue) lever. *n* = 18 couples. *p < .05, ***p < .001. (For interpretation of the references to colour in the Figure, the reader is referred to the web version of this article).

All behavioral data obtained in the P/E or M/D phase from session 6 onwards was averaged to contribute one P/E or M/D data point per female, respectively, and used for further analysis. In both phases of the estrous cycle, number of lever presses on the inactive lever was lower than lever presses on the BR and OR lever, but unlike in males, there was no difference in lever presses between the BR and OR lever (Levers *F*(2102) = 78.46, *p* < .001, *ƞ_p_^2^* = .61, Estrous cycle *F*(2102) = 0.35, *p* = .55, *ƞ_p_^2^* = .00, Levers*Estrous cycle *F*(2102) = 0.39, *p* = .68, *ƞ_p_^2^* = .01, Inactive vs BR and OR *p* < .001, BR vs OR *p* = .55, Fig. 3C). In other words, there was no significant pro-social choice (Fig. 3A) in both the P/E phase (*t*-test against 50 %: *t*(17) = -0.75, *p* = .46, d_z_ = -0.18), and the M/D phase (*t*(17) = -0.23, *p* = .82, d_z_ = -0.05). Interestingly, in the P/E phase, females completed fewer trials and thus earned less rewards compared to the M/D phase (*t*(17) = -3.95, *p* = .001, g_av_ = 0.98, Fig. 3B), but this did not result in a different BR preference (P/E vs M/D *t*(17) = -1.40, *p* = .18, g_av_ = 0.12, Fig. 3A). The order of testing (i.e. first as test rat or partner rat) did not significantly affect BR preference in either phase of the cycle (proestrus/estrus: *t*(16) = 2.0, *p* = .063, g_av_ =0.95, metestrus/diestrus: *t*(16) = 1.67, *p* = .11, g_av_ = 0.75), although substantial effect sizes were found for higher BR preference in the animals that were tested first (mean ± SEM for first as test rat: *M* = 57.4 ± 7.7 and *M* = 58.3 ± 6.4 versus first as partner rat: *M* = 36.4 ± 7.1 and *M* = 41.1 ± 7.6 for pro-/estrus and meth-/diestrus respectively).

There was no relation between bodyweight difference within a pair and BR preference (*r* = 0.50, *p* = 0.21. Mean ± SEM bodyweight for test rats: *M* = 226.9 ± 1.9 and partners: 227.3 ± 2.0, body weight difference range: 92.7–107.8 %). None of the body weight loss measures were correlated with BR preference (weight loss until start of testing: test rats *r* = -0.01, *p* = 0.99 and partners *r* = 0.44, *p* = 0.28; weight loss during FR3: test rats *r* = 0.09, *p* = 0.73 and partners *r* = −0.02, *p* = 0.94).

## 4 Discussion

In this study we fine-tuned a task to measure social decision making in rats and propose a fully automated, operant version of the pro-social two-choice task. Using an operant set-up with lever pressing, we successfully implemented a paradigm in which rats make a choice between earning a food reward for just themselves, or themselves and their partner. Although there was a lot of individual variation, we show that overall in males there is a preference for the option that yields a reward for both rats, suggesting that rats are sensitive to food reward delivery to a conspecific. Females do not show an overall preference for the pro-social option, regardless of differences in circulating hormone levels during the estrous cycle. Animals that were housed in a complex environment from postnatal day 26 onwards also did not show an overall preference for pro-social lever pressing. Costly prosocial behavior diminished pro-social choice.

### 4.1 An adapted, fully automated, operant pro-social two-choice task

The fact that we use an operant paradigm to test pro-social decision making is both a strength and a limitation. By reducing the very complex process of social decision making to an operant two-lever choice task, there is little face validity for real-life social dilemmas. It would be very difficult, if not impossible to mirror real-life rodent social dilemmas. However, this was not our goal. We aimed to study -in a tightly controlled setting- if rats attribute value to vicarious rewards. Part of the controlled setting was that we only tested pairs of rats that were familiar to each other. Given the fact that the percentage of BR choices was above chance, we conclude that male rats tested under these conditions indeed attribute value to vicarious rewards.

The design of this task is based on a chimpanzee pro-social two-choice task from Silk et al. (2005) and two rodent adaptions of this task (Hernandez-Lallement et al., 2015; Márquez et al., 2015). Despite differences in protocols, the underlying concept of a choice between two options is similar across studies and allows for the quantification of pro-social preferences for food sharing with conspecifics, in multiple species. There is much variation in the reported pro-social preferences and we are only beginning to touch upon the many factors that influence this social decision making process (Cronin, 2012; House et al., 2014; Marshall-Pescini et al., 2016). Interestingly, the majority of studies that report a preference for the pro-social option -including the current- show levels just above chance and the variability between individual animals is high. Here we report that male rats on average show a 60 % preference for the pro-social BR lever. This preference remains when the effort to obtain food is increased (from FR3 to FR5). However, as shown in the FR5-Costly protocol, the introduction of a rather subtle cost is enough to override the added value of a shared reward on the BR side. These observations seem to indicate that rats do show pro-social, but not necessarily altruistic behavior in this task.

The range of pro-social preference is in line with two other rat pro-social choice studies in males reporting a 55 % preference (Hernandez-Lallement et al., 2015) and up to 70 % preference for the BR choice (Márquez et al., 2015). In contrast to our automated, double-box design, both studies used a double T-maze design. Hernandez-Lallement and colleagues (2015) ruled out local enhancement as underlying mechanism to explain the preference, because the test rat always entered the choice compartment before the partner entered the corresponding compartment in the opposite maze. Márquez and colleagues extensively studied the ability of the partner to influence the choice of the test rat by training the partner in the opposite maze to actively display a preference for the BR side (Márquez et al., 2015). Partners would signal their preference for the BR compartment by nose poking the BR entrance port (explained as food seeking behavior) before the test rat made a choice. Test rats only showed a preference for the BR side if the partners were allowed to display their preference, showing that the cues related to reward delivery to the recipient rat, such as the sound of the pellets dropping, or chewing by the recipient, were not sufficient to drive prosocial choices. This display of the partner rat may explain the 65–70 % preference for the BR side, which is higher than the 60 % we obtained. This kind of signaling was not available to our recipient rats, and although we slightly food-deprived the rats (in contrast to the sated rats in the T-maze study by Márquez et al.), recipient rats in our study were apparently not able to adequately guide the test rat to obtain higher levels of both reward choice.

A possible confound in our study is the selection we made on boldness, selecting the least bold animals to act as test rats in the 2-choice task. The reason for this selection was a liberation task that was performed after the 2-choice task (data not reported in this paper), for which the most bold animals were chosen as test rats, as described in the original report on this task (Bartal et al., 2011). There is reason to believe that boldness might be an important personality trait in tasks with a quite stressful and demanding context (Sih and Del Giudice, 2012; Stamps and Groothuis, 2010); conversely, boldness may be less of an issue in less stressful tasks such as the 2 -choice task where there is no physical constraint. To what extent this assumption is true, however, is still open to debate. Firstly, less bold -compared to bold- animals might be more inclined to adapt their behavior to others, specifically in novel environments (Kurvers et al., 2011). The more extensive signaling opportunities the partner rats have in some set-ups, in terms of actively demonstrating their preference as mentioned earlier, might have been able to guide behavior of the test rat more strongly in our task. Of note, we extensively habituated the animals to the 2-choice task, so novelty may not play a major role during the actual test phase and the task does not demand high risk taking choices. We also did not find a correlation between bodyweight differences within a pair (as a proxy for dominance) and BR preference, so if boldness would indirectly affect dominance status through carryover effects on aggressiveness (Sih and Del Giudice, 2012), this is not likely to have had a major impact in our task. Secondly, a recent meta-analysis showed that bold animals are faster learners, but only when boldness is measured in response to a (potential) predator and not when it is measured by exposure to a novel object or exploration boldness (Dougherty and Guillette, 2018), the latter being more comparable to our boldness test. Despite these two arguments we cannot exclude a potential confound in our results due to selection on boldness. Therefore, careful consideration of the current data set in the light of the animals’ boldness is warranted.

We suggest that emotional contagion might be a possible mechanism underlying the pro-social behavior in this paradigm. Recently, it has been shown that observation of delivery of a food reward to a conspecific can lead to increased dopamine release from the nucleus accumbens core (Kashtelyan et al., 2014) and a similar effect was found after the emission of 50 kHz affective calls, but not 22 kHz alarm calls, by a conspecific (Willuhn et al., 2014). This increased dopamine and affective vocalization were present at the first trial only and might have signaled vicarious reward learning, maybe anticipating their own reward. If the rewarding sensation of receiving a food pellet is transferred from the partner to the test rat (for example via visual communication or via an increased emission of affective calls by the partner) and likewise induces an experience of reward in the test rat, the value attributed to the BR lever might be higher than the value attributed to the OR lever, which would result in a preference for the BR side. Comparable operant choice experiments have been performed in the addiction field, aimed specifically at studying preferences and the difference in value attribution between different types of rewards (for example, drugs of abuse versus food (Schwartz et al., 2017). As the contribution of vicarious rewards in this type of paradigm is still highly speculative, it would be of interest to measure dopamine signaling during BR and OR choices. Moreover, measuring ultrasonic vocalizations might shed more light on the communication between test rat and its partner in the context of emotional contagion.

In general, in both humans and non-human animals, females are usually found to be more empathic and show more pro-social behavior than males in a variety of different empathy related behaviors (Christov-Moore et al., 2014). In rodents, a few studies that tested both males and females showed that females acted either equally empathic or more empathic than males (Bartal et al., 2011; Han et al., 2020; Langford, 2006; Langford et al., 2010). More specifically, it was reported that mice show emotional contagion in the form of an increase in pain behavior, i.e. writhing, in proximity of a conspecific that experiences pain. Both males and females showed this in proximity of a familiar conspecific (cage mate or sibling); yet only females also showed this behavior in proximity of a stranger (Langford, 2006) and females approached a conspecific in pain more readily than males (Langford et al., 2010). In rats, similar levels of emotional contagion were found in males and females by measuring freezing behavior after seeing a conspecific receiving a shock (Han et al., 2020). However, a larger proportion of female rats, compared to males, were willing to liberate a trapped cage mate that was stuck in a cylinder, and they were also faster in doing so (Bartal et al., 2011).

Based on these studies we hypothesized that females would show similar or potentially higher average levels of pro-social behavior than males. To our surprise, females did not show a preference for the BR side. A hypothesis could be that females tend to show more pro-social behavior under stressful or painful circumstances. Indeed most of the rodent studies report heightened pro-sociality in females with experimental paradigms that introduce either pain or stress. Moreover, a recent study found that female rats were able to show pro-social behavior in the form of consolation after witnessing a fight between conspecifics (Heinla et al., 2020). Taking into account that pro-social behavior in rodents seems to be more prominent when there is an affective motivation (Bartal et al., 2016), it could be proposed that females might be more sensitive to affective cues. However, further research is necessary to draw any firm conclusions. Another parameter that could affect the behavior of the female rats is that individual animals acted both as the test rat and as the partner on separate occasions, whereas in males individual animals only acted as test rat or partner rat. There are studies that link pro-social behavior with reciprocity, suggesting that animals show pro-social behavior following the simple rule “help if you have received help” (Taborsky et al., 2016). It could then be hypothesized that females who had already been included as partner rats were less inclined to choose the BR lever as test rats, because they had not always received a pellet when they were acting as partners. However, we found no significant differences in BR preference between females tested first as partner rats and those first tested as test rats. Indeed, rats seem to reciprocate help mainly based on their most recent encounters, instead of integrating social experience over longer timespans (Schweinfurth and Taborsky, 2020).

Given the influence of fluctuating female sex hormones on social behavior in both humans (van Wingen et al., 2011) and rodents (Ervin et al., 2015), we furthermore hypothesized that the percentage of BR choices might be susceptible to hormonal changes across the estrous cycle. However, no differences were found between preference in the P/E versus the M/D phase (with high and low levels of progesterone and estradiol, respectively). Interestingly, females in the P/E phase completed fewer trials and thus earned less rewards compared to M/D, but this did not affect BR preference. This is in accordance with a recent study in females on the effect of circulating hormone levels on reinforcement learning, where females in the P and E phases completed fewer trials compared to the M/D phase (Verharen et al., 2018).

### 4.2 Influence of complex housing

We next studied the influence of rearing animals in a complex housing environment on social decision making. Complex housing provides rats with physical and social enrichment and cognitive stimulation in the form of novelty (i.e. a maze on the top floor that is changed every week). Previously our lab showed that complex housed males displayed more social play with unfamiliar peers in adolescence, but in adulthood showed less social interest towards an unfamiliar peer (Kentrop et al., 2018); however, neither protocol allowed selection of a pro-social choice. Therefore, we reverted to the present paradigm to study this aspect. Since pro-sociality and emotional contagion are known to increase with increasing levels of familiarity (Preston and de Waal, 2001), sharing food with a -familiar- cage mate in the pro-social two-choice task is likely different from encounters with unfamiliar peers.

Our results indicate that towards familiar animals, as currently measured, complex housed males on average do not act pro-socially. This is based on differences from chance preference, which was significant for standard housed but not for complex housed animals. It should be noted, however, that there are large individual differences and a subset of animals in the complex housed group actually acted pro-socially. If we distinguish between animals as either pro-social, indifferent, or not pro-social, the complex housed animals were found to be either prosocial (52 %) or not-prosocial (48 %), while the standard housed animals were prosocial (64 %), indifferent (18 %), or not prosocial (18 %) (Fig. S2). This is suggestive of a larger number of non-prosocial animals in the complex housed group. Thus far, it has been widely reported that environmental enrichment during early life or adolescence enhances social behavior and has anxiolytic effects (Brenes et al., 2006; Sparling et al., 2018). In that respect we did expect augmented pro-social behavior in the CH animals compared to SH animals. However, according to Bartal et al. (2016), rats that were less stressed were also less inclined to show pro-social behavior. In our paradigm the rats remained in the complex housing condition throughout the experiment and we have reported earlier that these rats become quite active, quickly habituate to new situations, and -importantly- have diminished behavioral control and are less interested in conspecifics (Kentrop et al., 2018; van der Veen et al., 2015). Because the complex housed rats experience continuous exposure to many peers, they might be more used to ignore their vocalizations and communication signals and as a result be less attentive towards them in the task. We were not able to take into consideration the more mixed hierarchical relationships complex housed animals develop among them; a parameter that could also affect their behavior. Noteworthy, direct comparison of both housing conditions did not reveal changes. This might be related to the high variability between individual animals and more polarized behavior in the complex housed group, which warrants further investigation and higher sample sizes than presently used, despite the fact that our groups sizes in experiment #2 were already quite high for an explorative study (62 pairs in total).

### 4.3 Concluding remarks

We conclude that the fully automated, operant two-choice task presented here is suited to measure pro-social decision making in rats. The average BR preferences in males are in line with earlier published pro-social two-choice experiments conducted in rats and other species. The task is sensitive enough to measure changes in behavior as a result of (subtle) manipulation of experimental contingencies, allowing to study in future biological factors underlying social decision. Complex housing conditions might impact on pro-social interaction with familiar conspecifics, but results should be interpreted with caution given the high variability between individual animals. Moreover, additional experiments are required in order to determine the effect of starting complex housing in adolescence versus acute effects of complex housing. It would be interesting to look into how factors such as familiarity, reciprocity, and sex of partner may affect the BR preference of the rats in this task.

## Declaration of Competing Interest

The authors declare that they have no known competing financial interests or personal relationships that could have appeared to influence the work reported in this paper.
