# Supplementary material for: Pro-social preference in an automated operant two-choice reward task under different housing conditions: Exploratory studies on pro-social decision making
Source: Dev Cogn Neurosci. 2020 Jul 18;45:100827. doi: 10.1016/j.dcn.2020.100827 (PMC7393525; doi:10.1016/j.dcn.2020.100827)
Supplement: Supplementary file 1 [file mmc1.docx]

Supplementary figures

Figure S1: Pro-social Both Reward (BR) preference in males for combined pilot experiments (A) BR preference on a free choice FR3 protocol represented as the percentage of BR choices per session. Preference for the BR lever at a group level was stable over time (F(7,238) = 0.20, p = .91, ƞp2 = .01). The last 3 sessions were averaged for (B) BR preference per individual animal, *indicates a significant difference from 50% chance on a group-level (*t*-test against 50%: *t*(34) = 2.29, *p* = .03, *d*_z_ = 0.39). (C) Number of rewards earned per session. Male rats optimized performance over the course of free choice FR3 testing, as seen by an increase of rewards obtained by the test rats (F(7,238) = 7.06, p < .001, ƞp^2^ = .17). (D) Number of responses on the inactive (grey), OR (pink), and BR (blue) lever. The number of lever presses on the inactive lever was significantly lower than on the BR and OR lever and presses on the BR lever were higher than on the OR lever (F(2,102) = 57.70, p < .001, ƞp^2^ = .53, Inactive vs BR and OR p < .001, BR vs OR *p* < .001). *n* = 35 couples. *p<.05, ***p<.001.


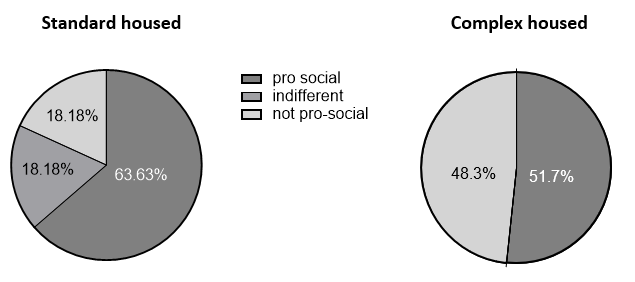


Figure S2: Categorization of animals according to their BR preference in the standard and complex housing conditions. In the graphs the percentage of rats that were categorized as pro-social, indifferent or not pro-social in the FR3 condition is depicted. Construction of categories: BR preference (mean of the last 3 days on the FR3 protocol) was plotted for each animal with their individual SD over these 3 days; if the upper or lower SD bound did not cross the 50% line, an animal was considered either pro-social or not pro-social, if the SD did cross the 50% line, the animal was considered to be indifferent.
